# OP04 Patient Preferences Regarding Pharmaceutical Services: The Ukrainian Experience Of MaxDiff Analysis

**DOI:** 10.1017/S0266462325100639

**Published:** 2025-12-29

**Authors:** Tamara Mahanova

## Abstract

**Introduction:**

Incorporating patient preferences into health technology assessment represents a promising avenue for its enhancement. However, the complexity and diversity of methods for evaluating preferences, coupled with uncertainties regarding their impact on decision-making processes, present significant challenges to their effective integration. Consequently, the objective of this study was to evaluate the application of the MaxDiff analysis methodology for determining patient preferences, with a focus on pharmaceutical services as a case study.

**Methods:**

The experimental design for scaling MaxDiff was developed using Sawtooth Lighthouse software. The design parameters included 12 pharmaceutical services. Descriptive statistics, hierarchical Bayesian analysis, latent class analysis, and logistic regression methods were employed to generate preference results.

**Results:**

The most preferred services were identified as follows: the creation of a personalized list of safe medications to address patient needs, with a quantitative contribution of 0.5; and the provision of rapid tests (for influenza and *Helicobacter*) and stroke risk assessment (quantitative contribution of 0.31). In contrast, patients did not prioritize services related to contraception selection or weight control and weight loss program development. Descriptive statistics, hierarchical Bayesian analysis, and logistic regression methods sequentially identified the most preferred services. Latent class analysis revealed two distinct consumer segments: Segment 1 (39%) and Segment 2 (60%), which differed in their sociodemographic characteristics and preferred services.

**Conclusions:**

The obtained results can be utilized as patient-based evidence to understand patients’ unmet needs in the assessment of technologies such as pharmaceutical services, taking into account the local context. Despite the simplicity of the MaxDiff analysis methodology, the resource intensity and the level of knowledge among specialists and patients regarding preference collection methods remain significant barriers to its implementation.

